# Potential future malaria transmission in Odisha due to climate change

**DOI:** 10.1038/s41598-022-13166-5

**Published:** 2022-05-31

**Authors:** Ruchi Singh Parihar, Prasanta Kumar Bal, Atul Saini, Saroj Kanta Mishra, Ashish Thapliyal

**Affiliations:** 1 Centre for Atmospheric Sciences, Indian Institute of Technology Delhi, New Delhi, India; 2 Qatar Meteorology Department, Civil Aviation Authority, Doha, Qatar; 3 Graphic Era Deemed to be University, Dehradun, Uttarakhand India; 4 Delhi School of Climate Change and Sustainability, Institution of Eminence, University of Delhi, Delhi, India

**Keywords:** Climate sciences, Climate change, Climate-change impacts, Environmental health

## Abstract

Future projections of malaria transmission is made for Odisha, a highly endemic region of India, through numerical simulations using the VECTRI dynamical model. The model is forced with bias-corrected temperature and rainfall from a global climate model (CCSM4) for the baseline period 1975–2005 and for the projection periods 2020s, 2050s, and 2080s under RCP8.5 emission scenario. The temperature, rainfall, mosquito density and entomological inoculation rate (EIR), generated from the VECTRI model are evaluated with the observation and analyzed further to estimate the future malaria transmission over Odisha on a spatio-temporal scale owing to climate change. Our results reveal that the malaria transmission in Odisha as a whole during summer and winter monsoon seasons may decrease in future due to the climate change except in few districts with the high elevations and dense forest regions such as Kandhamal, Koraput, Raygada and Mayurbhanj districts where an increase in malaria transmission is found. Compared to the baseline period, mosquito density shows decrease in most districts of the south, southwest, central, north and northwest regions of Odisha in 2030s, 2050s and 2080s. An overall decrease in malaria transmission of 20–40% (reduction in EIR) is seen during the monsoon season (June-Sept) over Odisha with the increased surface temperature of 3.5–4 °C and with the increased rainfall of 20–35% by the end of the century with respect to the baseline period. Furthermore, malaria transmission is likely to reduce in future over most of the Odisha regions with the increase in future warm and cold nights temperatures.

## Introduction

The assessment of vector-borne diseases, such as malaria, transmission under future climate change in terms of the changes in temperature and rainfall is important as the human health is at risk, particularly changes in the frequency of malaria transmission^1^. Numerous studies shown that the climate change is likely to expand the distribution of various vector borne diseases such as malaria and dengue in higher altitudes and higher latitudes regions across the globe^2–6^. Many of the prior studies suggest that the changes in the distribution of malaria is partially due to the anthropogenic climate change^7–13^. For example, malaria cause of morbidity and mortality in south and southeastern Asia is likely aggravated by climate change^14,15^. Conversely, few other studies suggest that the changes in malaria transmission over Europe and USA is due to the effect of non-climatic factors and are due to social-economic development^16–18^. Gething et al.^19^ highlighted that the warming temperature in future due to climate change affects the changes in malaria transmission. To understand the impact of global warming on Indian monsoon, many past studies have used various climate models based on different scenarios of emission of greenhouse gases to assess future monsoon climate in India. Results from many such studies show that rising greenhouse gas concentrations have resulted an increasing trends of temperature and rainfall during the monsoon season over India in future (Sandeep et al.^20^, Mishra et al.^21,22^, Chaturvedi et al.^23^).

In this context, few studies suggest that climate change has a major impact on changes in vector borne diseases, specifically, changes on malaria transmission in different parts of the world^24,25^, Sarkar et al. ^26,27^. Climate change has a direct influence on malaria transmission and an increase in the temperature and rainfall is more conducive for the breeding of mosquito and cause the increase in frequency and intensity of the malaria diseases^28–31^. Hence, there is an urgent need of understanding the possible characteristics of malaria transmission dynamics and its connection to climate change in the context of temperature and rainfall variabilities.

There are different dynamical models such as LMM_RO^32^, MIASMA^12^, VECTRI^33^, UMEA^34^, and MARA (Craig et al.,^35^) are being used over different regions in earlier studies to produce climate change impact assessments for malaria transmission. These models are driven by the outputs from different global climate models using different future scenarios. In this context, the studies on changes in malaria transmission in future over India due to the climate change are very limited especially, in the context of malaria transmission dynamics on a regional scale. Based on a recent study by^36^, state wise future malaria intensity is predicted using temperature and relative humidity data from a regional climate model for the projection period 2030. In another, study, a fuzzy-based malaria transmission model is generated over India using temperature and relative humidity data, obtained from CORDEX South Asia for baseline (1976–2005) period and for the projection period 2030s (2021–2040) under RCP 4.5 scenario^26^. The results from the above two studies indicate that the malaria intensity in some of the Indian states may increase by 2030s due to climate change. Again, in another study by Chaturvedi & Dwivedi (^37^), VECTRI model is simulated using temperature and rainfall data sets driven from five different global climate models for the future projection period 2006–2050 and they found an increase in future malaria transmission in most of the Indian region during both southwest and northeast monsoon months (June–October). However, VECTRI simulations using the outputs from one of the models show an overall decrease in the malaria transmission in some parts of the Indian region.

The primary aim of this research is to estimate the climate change impacts on malaria transmission using a dynamical malaria model, VECTRI driven by climate outputs from a global climate model CCSM4. Our results are focusing on a particular malaria prevalence region, Odisha, where the malaria transmission is of about 26.9% of the total malaria cases in India^38^. The model is run for the baseline/historical period 1975–2005 and for different future projection periods such as 2020s (2005–2035), (2005–2035), 2050s (2035–2065) and 2080s (2065–2095). This VECTRI model predicts the dynamics of malaria transmission over a particular region based on the effect of climate change, population density and surface hydrology in that region. Moreover, this study illustrates the potential impact of future climate change on the spatial and temporal distribution of malaria transmission in Odisha.

Odisha faces cyclones and floods with a regularity, almost in every year that significantly affect its economy. Therefore, climate change has direct impact on the distorted growth rates of social and economic sectors of Odisha. In Odisha, more than 80% of its population are living in rural areas and the monthly per capita consumer expenditure for Odisha is below the respective national averages^39^. Odisha contributes about 43.06% of total malaria deaths of the country^38^. Endemicity of malaria over Odisha, with highest malaria burden is seen over some of high elevated regions of Central and South Odisha regions, dominated by hilly areas along with some plains and forested areas including the districts Malkangiri, Koraput, Kalahandi, Rayagada, Nuapada, Nawarangpur, Boudh and Phulbani etc.^40,41^. Keeping this as economical and climatology background, it is thus very important to assess the future climate sensitivity of malaria prevalence in Odisha. Currently, limited studies are available over the Odisha region which focus climate modeling studies for the future climate change projections to drive any malaria health impact models. In this study, changes in the climatically suitable malaria prone districts in Odisha are identified based on the changes on the long term climate trends. Also, to ensure that climate change has a major impact on malaria transmission over Odisha region, our study focuses on a relationship between various aspects of climate change and malaria transmission at different spatial and temporal scales. In addition, this study estimates the impacts of future climate change on potential malaria transmission dynamics over Odisha on a local scale. We incorporate the future projections of climate estimates of temperature and rainfall in the malaria transmission VECTRI dynamic model at a regional scale under a transient climate change scenario RCP 8.5 and estimate the distribution of future malaria transmission.

The reminder of this paper has been organized as follows. "Study area" section provides the methodology details of the climate model, observation data sets and VECTRI model simulations used in this study. "Results" section shows the compressive results section and some concluding remarks with discussions are given in "Discussions" section.

## Study area

**Figure 1:**
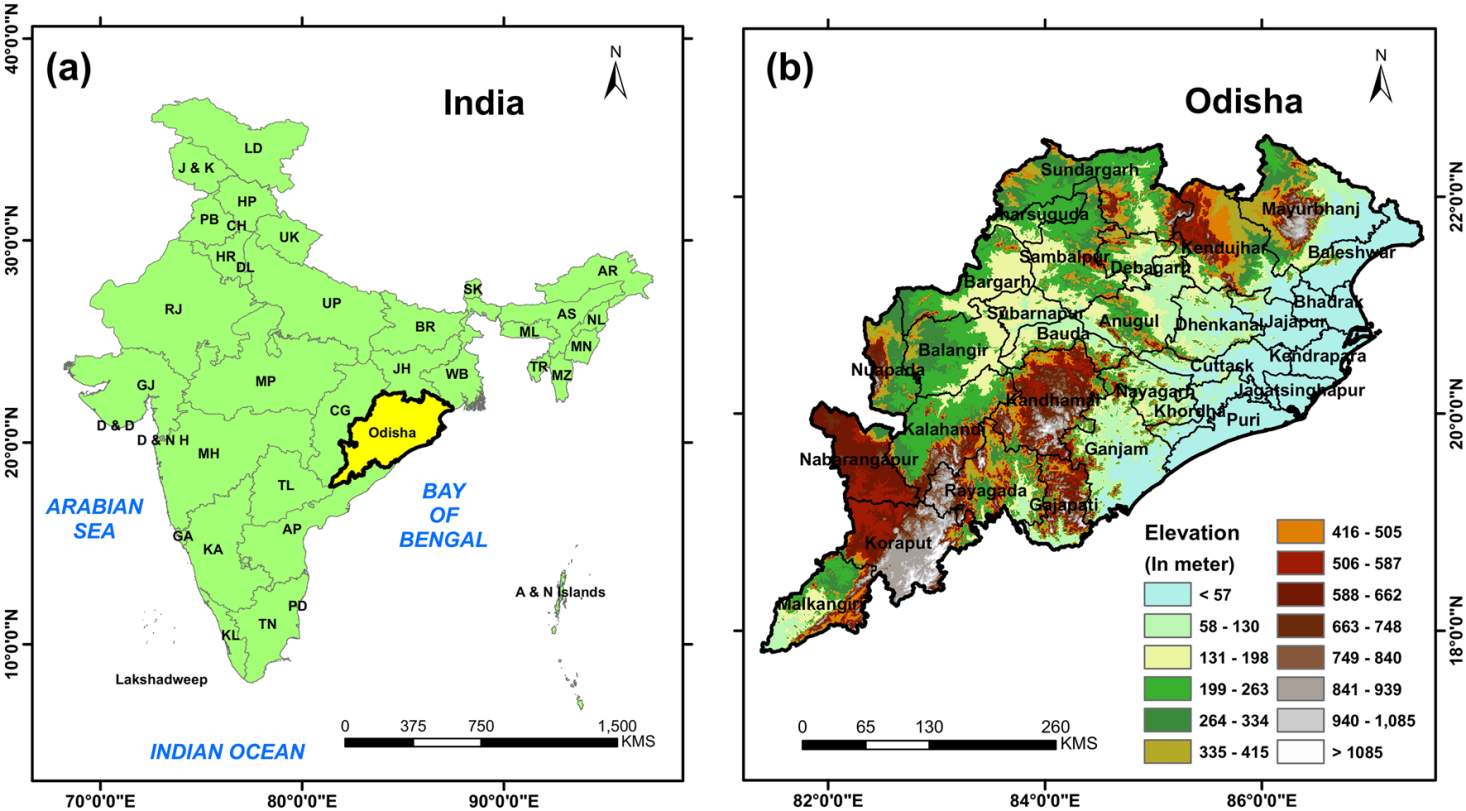
Study area that highlights Odisha (district wise distributions) and  elevation of all the districts from the sea level.

The Odisha state, lies between 17.7°N and 22.73°N latitudes and 81.37°E to 87.53°E longitudes is one of the most vulnerable regions in India which has been heavily affected by the changes of climate and extreme weather events such as cyclones, floods and heat waves. It is surrounded by the Bay of Bengal in East and West Bengal state in the North East, Jharkhand state in the North, Chhattisgarh state on the west and Andhra Pradesh state in the South. Due to its specific geoclimatic condition, this state gets affected by many climate induced natural disasters, which have become more frequent and widespread among all the 30 districts in Odisha. In general, most of the calamities such as cyclones, floods and heat waves occur mainly during March to October. It is located in the core monsoon zone of India and receives heavy rainfall during the summer monsoon season (June to September). The climatology over Odisha suggests that the maximum temperature exceeds 40 °C during the month of May and the annual rainfall is about 1451.2 mm, out of which about 75–80% of its annual total rainfall received during summer monsoon season (http://cesorissa.org/soe/Climate.pdf). In addition, during summer monsoon season, extreme climate events like heavy rainfall and flash floods cause the prevalence of both water-borne and vector-borne diseases such as Malaria, Dengue, Chikungunya, Cholera, Filariasis etc. The ambient temperature, heavy rainfall with a high humid climate in Odisha make favorable condition for the transmission of malaria in most of the regions of the state. The average population density of Odisha is 269 (Census, 2011) and dense population seen over the northeastern parts of the state. Further, the elevation of Odisha ranges from 1400 to 1700 m at the southern part (Deomali mountain is located near Koraput district at an elevation of 1672 m above sea level) and nearly less than 24 m and close to sea level in the coastal districts (Fig. 1).

### Data and methods

VECTRI model is used in this study to simulate the malaria dynamics in the form of vector development and the malaria transmission peaks during different seasons over Odisha. We have used daily input data for daily rainfall, temperature and human population density data for the whole Odisha (district wise distribution) in the experimental set up while running the dynamical model VECTRI**.** The model is simulated over Odisha to predict the dynamics of the malaria transmission of the *Plasmodium falciparum* parasite that is transmitted through the *Anopheles culicifacies* vector. From the previous studies, it is found that climate of Odisha is favourable for the P. falciparum parasite which is the major cause of malaria transmission in the state and An. *culicifacies* is likely to be considered as one of the major malaria vectors in the state which is prevalent throughout the year (Ranjit,^42,40^. Also, minimum temperature of 16.5 to 18 °C as the threshold temperature values required for the development of *Plasmodium falciparum* parasite^8^. Therefore, while simulating the model, the parameterization scheme is being modified according to the various climatic factors such as temperature and rainfall those are responsible for the survival of this particular malaria vector An. *Culicifacies* over Odisha. The list of parameters with the description and their values set up in the model are described in details with the VECTRI model architecture in the previous studies^43,33^. The observed daily mean, maximum and minimum temperatures of 1° × 1°^44^ and rainfall data of 0.25° × 0.25° horizontal resolution^45^ for the 30 years baseline period (1975–2005) are obtained from India Meteorological Department (IMD) for all the districts of Odisha and the data are being used as inputs to the VECTRI model for the simulation. Similarly, daily temperature and rainfall data (bias corrected) from the CCSM4 global climate model are obtained from the ESGF portal for the baseline period as well as for three future projection periods 2020s (2005–2035), 2050s (2035–2065), and 2080s (2065–2095). The population data is obtained from https://sedac.ciesin. columbia.edu /data/set/ popdynamics- global-pop-density- time-series-estimates. This population density grid time series estimates consist of the number of persons per 30 arc-second (~ 1 km) grid cell divided by the area of the grid cell in kilometers for each of the target years^46^. We have used population data and CCSM4 model data as inputs to the VECTRI model which are regridded to the same resolution before running the model. From the various output parameters generated by the VECTRI model, the following few variables are considered for our analysis: rainfall (mm day^-1^), mean temperature (°C), mosquito density or vector density (per square meter) and entomological inoculation rate (EIR) in number of infective bites per person per day(i/b/d). VECTRI outputs from CCSM4-simulations for mean temperature, annual rainfall, vector density and EIR are compared with those of the VECTRI outputs from the IMD observed values during the baseline period to evaluate the performance of the VECTRI model. The climate change assessments with respect to the temperature and rainfall change and the corresponding change in vector density and EIR have been estimated by comparing baseline data with future data generated from the VECTRI model. In addition, Bias, Root Mean Square Error(RMSE), correlation and error between VECRI model outputs forced with IMD observation and VECRI model outputs forced with CCSM4 climate model for all the parameters (temperature and rainfall with vector density and EIR) are computed to assess the performance of the VECTRI model. In general, the analyses include model validation, monthly and seasonal distributions for the future projections period 2020s 2050s and 2080s to predict the potential future change in dynamics of the malaria transmission over Odisha. Since malaria transmission is largely seasonal corresponding to rainy season and maximum number of malaria cases and deaths occur during the monsoon seasons in India^47^, we have focused future malaria transmission dynamics during June to September, JJAS and October to December, OND seasons in our analyses. Warm nights are being computed using the 90th percentile of the minimum temperature time series data sets during the monsoon season (JJAS) for historical period as well as for the three projections period over the whole Odisha on a spatial scale. Changes in both warm and cold nights are represented in terms of temperature changes in 2020s, 2050s and 2080s when compared to the historical period. On the similar way, cold nights are computed using the 10th percentile of minimum temperature time series data during the winter season (OND) for the same period. Then changes in EIR is being computed for only those warm nights and cold nights period during 2020s, 2050s and 2080s with respect to the historical period to estimate the future malaria transmission dynamics in the changing extreme temperature events in future.

## Results

### Model validation

The IMD observations and CCSM4 model outputs of daily maximum & minimum temperatures and rainfall for the historical period 1975–2005, used to force the VECTRI model, and the model outputs are inter-compared to understand the reliability of the CCSM4 model.

**Figure 2:**
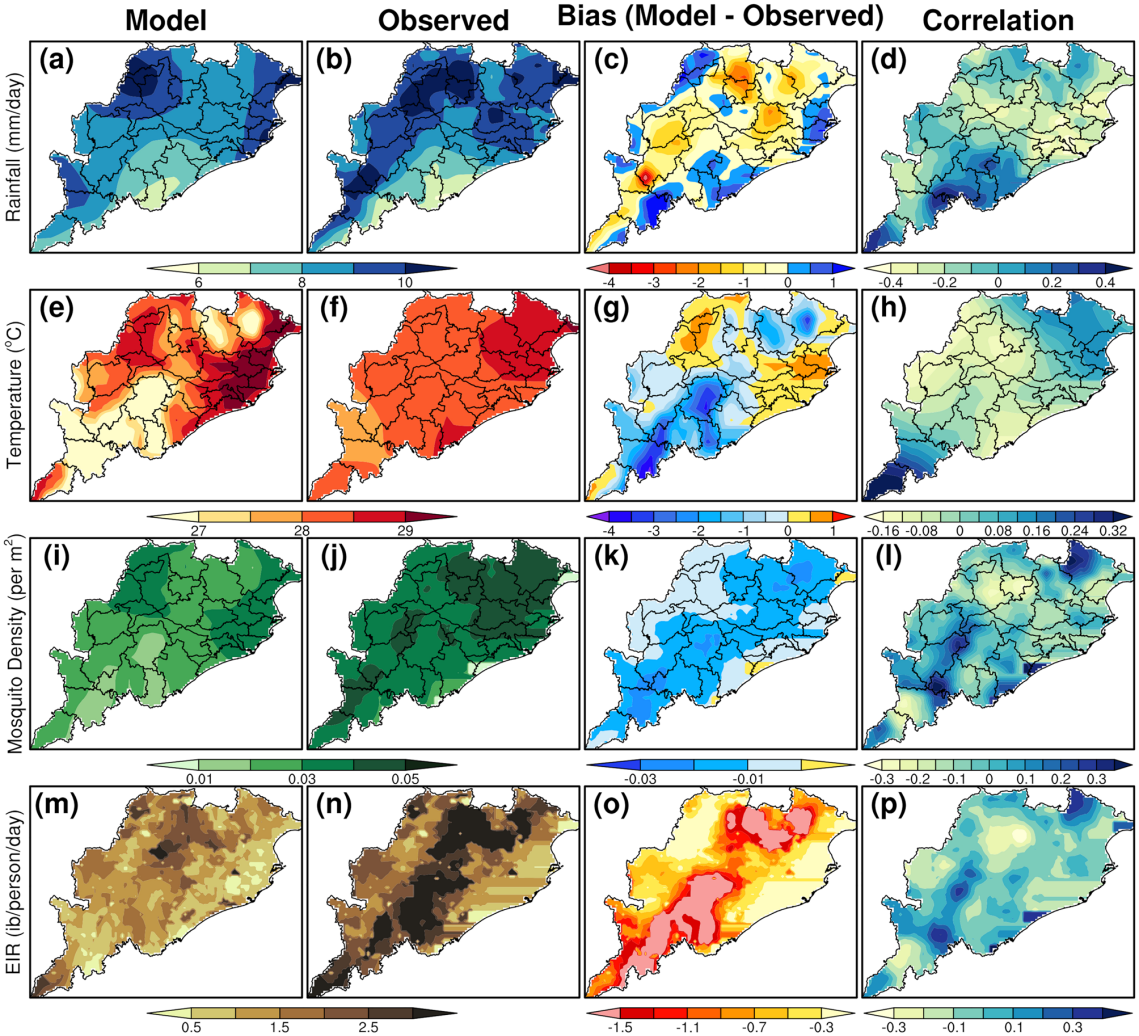
CCSM4 model evaluation with respect to the IMD observation during the pariod 1975–2000 (June to September).

**Figure 3:**
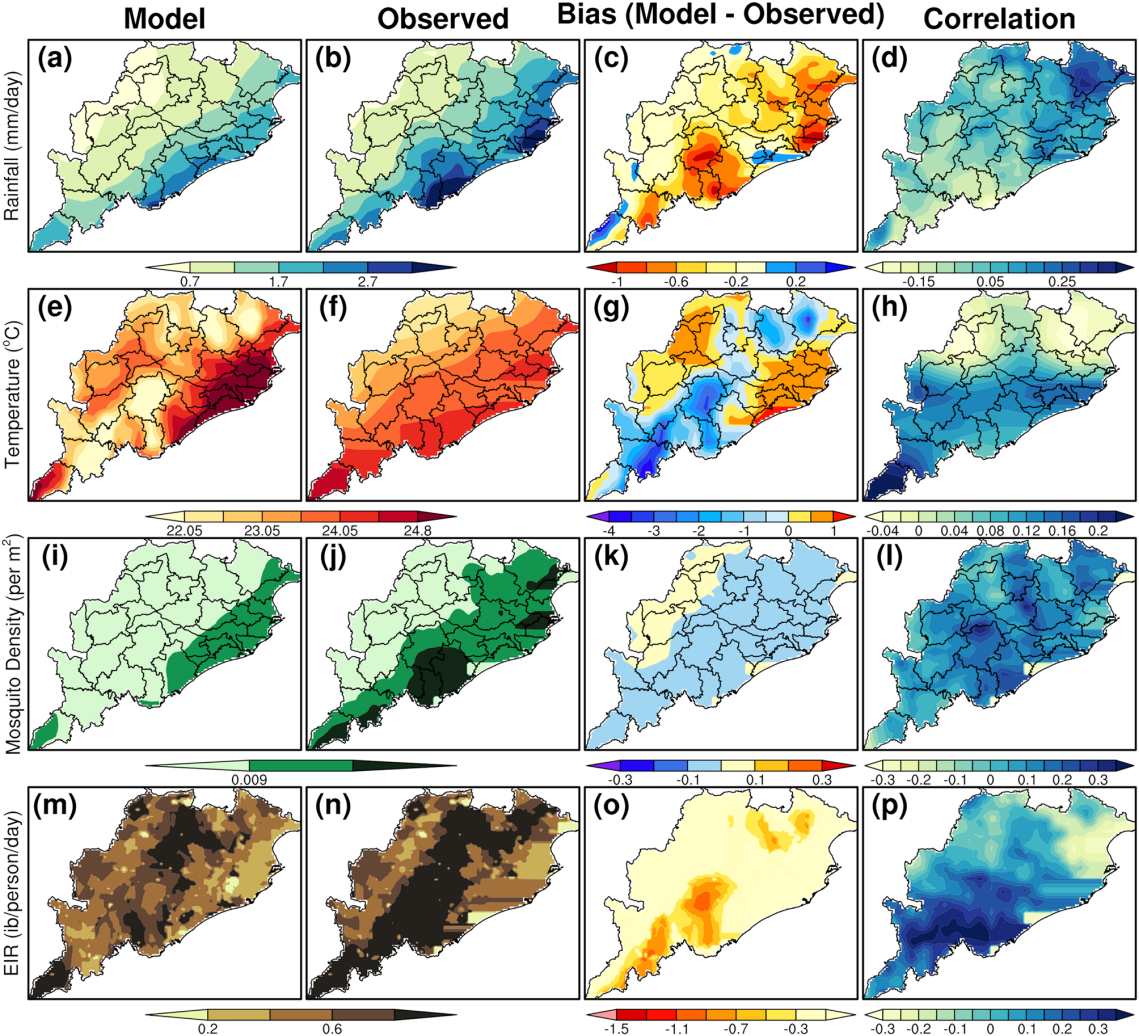
CCSM4 model evaluation with respect to the IMD observation during the period 1975–2000 (October to December).

The spatial pattern of mean surface temperature, rainfall, vector density, and EIR from the VECTRI model using CCSM4 model and IMD observations qualitatively shows similar patterns over Odisha. Though the vector density and EIR from CCSM4 forced VECTRI are underestimated as compared to IMD forced VECTRI, the spatial pattern of mean rainfall and temperatures are in quite reasonable with minimal biases between the simulations during both the monsoon seasons (Figs. 2 and 3). Specifically, during JJAS period, the CCSM4 model shows good skill compared to IMD observations with a slight dry bias of 1 to 4 mm/day in rainfall over most of the Odisha region, cold (warm) bias of 0.5–3 °C (0–1 °C) in southern and northern parts (coastal and western districts) of Odisha.

Further, the VECTRI model forced with CCSM4 model shows a negative bias in the vector density (0.03 per sq. m) and EIR (0.3–1.5 infective bites/persons/day and above in few places) over most parts of Odisha compared to the VECTRI outputs forced with IMD observations during the JJAS period. It can be further noticed that the negative biases of vector density and EIR are minimal during the OND season when compared to JJAS season. According to Tompkins & Ermert^33^, if EIR values are above 0.01, then that area will be more favorable for the malaria transmission and if EIR values are, less than 0.01, then no transmission occurs in that area. EIR is directly affected by the vector density and the number of humans in a given local situation and the duration of sporogonic cycle of the mosquito vector and all these factors are sensitive to the changes in temperature and rainfall^48,49^, get reduced with increase in temperature. From our results, it is very clear that, EIR values are above 0.05 in almost all the regions in Odisha that indicates the higher intensity of the malaria situation in the state.

Again, for the case of temperature, the CCSM4 forced model simulation shows a warm bias of 1 °C over western and eastern parts of the state that is quite lesser compared to the warm bias estimated during the JJAS season. Further, results from CCSM4 forced simulations shows a dry bias of 1 mm/day of rainfall over most parts of Odisha when compared with the IMD forced rainfall simulations.

The bias, RMSE and model errors (%) of temperature, rainfall, vector density and EIR averaged over Odisha between the VECTRI model outputs forced with CCSM4 and IMD are given in Table 1. The RMSE values for rainfall are assigned in mm/day. VECTRI model errors forced with both observation and CCSM4 for all the 4 parameters such as temperature, rainfall, vector density and EIR are about 2.99%, 10.64%, 34.8%, and 35.6%, respectively. The magnitude of biases and RMSE for all the parameters show a relatively smaller bias.

**Table 1:** Model validation statistics for the period 1975–2005. Model experiments forced by the inputs derived from IMD observations and bias corrected CCSM4 model are considered for estimating the statistics.

Parameters	Bias	RMSE	Error (%)
T2M	− 0.7912	1.3039	2.99
Rain fall	− 0.4131	3.0822	10.6465
EIR	− 0.1639	0.3095	35.6
Vector	− 0.0109	0.0196	34.8

*Negative sign indicates CCSM4 driven parameters underestimate than that of IMD.

### Future projections of malaria transmission due to climate change

Table 2 shows the percentage change in annual rainfall, mosquito density and EIR for the projection periods 2020s (between 2005 and 2035), 2050s (between 2035 and 2065) and 2080s (between 2065 and 2095) using CCSM4 compared to the baseline period (during 1975–2005). It is found that an increase of about 3%, 11% and 17% in annual rainfall for the periods 2020s, 2050s, and 2080s, respectively. The EIR (vector density) values show a general decrease (increase) of 16% (18%), 29% (32%), and 37% (36%) for the same projection period compared to the baseline period. This indicates that the intensity of malaria transmission in future may potentially decrease with increase in rainfall. The increase in larvae density with the decrease in malaria transmission indicates that the VECTRI model includes a representation of flushing effect of early stage of larvae with the increase in rainfall and these results are in well agreement with few previous studies^50^.

**Table 2:** Percentage of projected change with reference to the historical period (1975–2005).

Parameter	2020s (%)	2050s (%)	2080s (%)
Rain Fall	3.38845760267612	11.1625308644294	17.4760479777255
EIR	− 16.5072630443462	− 29.3339223344146	− 37.5566745548705
Vector	18.7296871771435	32.8697103549986	36.8516360749373

#### Monthly projections

**Figure 4:**
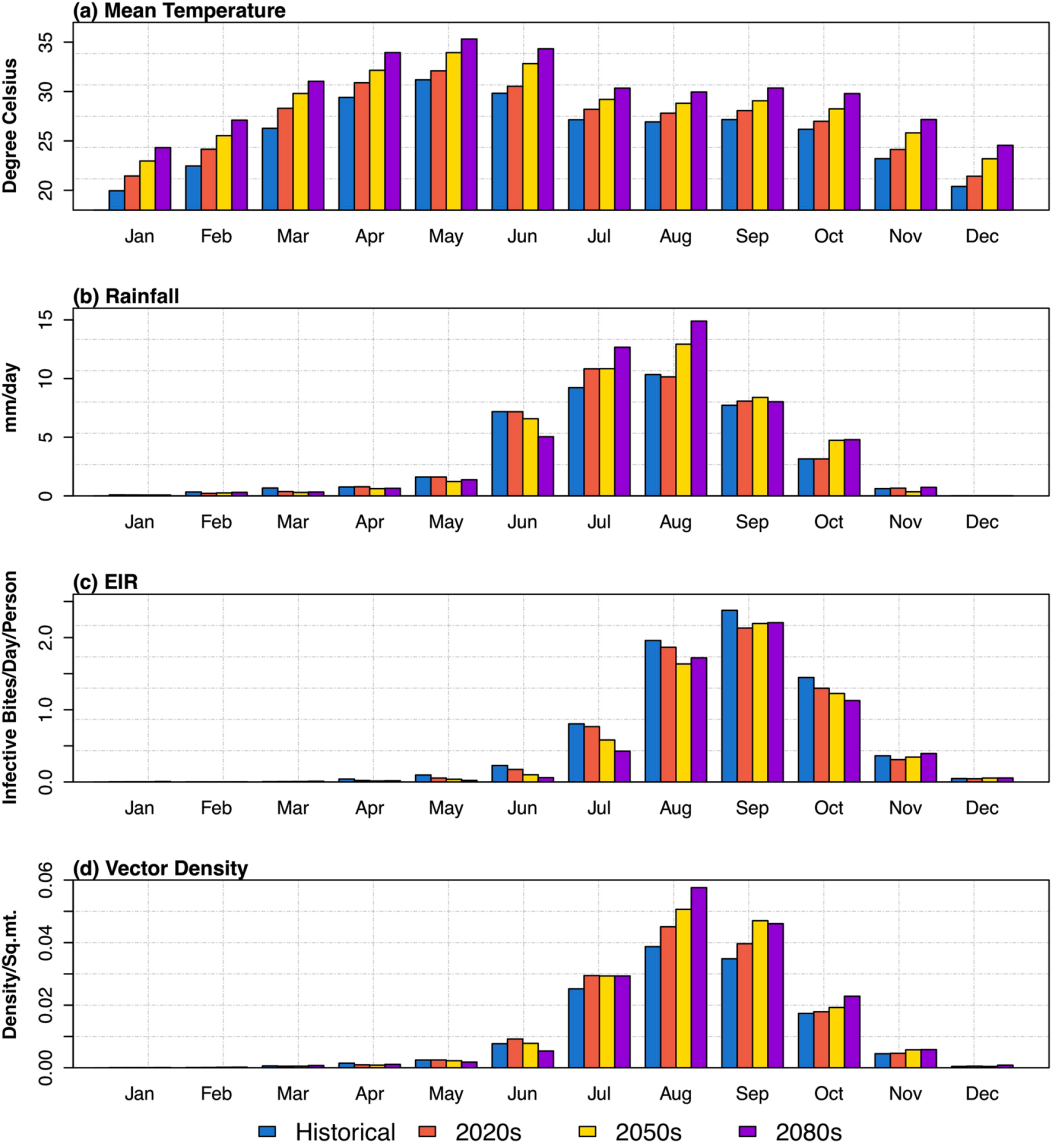
Monthly mean values of simulated parameters over the whole Odisha correspond to four time slices (Historical, 2020s, 2050s, 2080s).

Figure 4 shows the future projections in each month of temperature, rainfall, EIR, and vector density averaged over Odisha. In general, the model predicts an increase in the mean temperature (of about 3–4 °C by end of the century) and rainfall (of about 20–60% by end of the century) during both the monsoon and post monsoon seasons with reference to baseline period. In particular, an increase in the daily rainfall during the southwest monsoon (June–September) seen for all the projection periods i.e. for 2020s, 2050s and 2080s compared to the baseline period. The monthly distribution of malaria transmission in terms of vector density and EIR follows the rainfall patterns over Odisha. The peaks in the vector density and EIR during the months August to September follows the peaks in rainfall. It is interesting to note that during monsoon and post-monsoon months of baseline period, the vector density and EIR values show higher, suggesting higher transmission rates of malaria in a monsoon environment. However, the mean monthly variability of vector density (EIR) for the projection periods 2020s, 2050s, and 2080s shows an increase (decrease) from the month of June to October compared to the baseline period.

#### Projections during summer monsoon season

**Figure 5:**
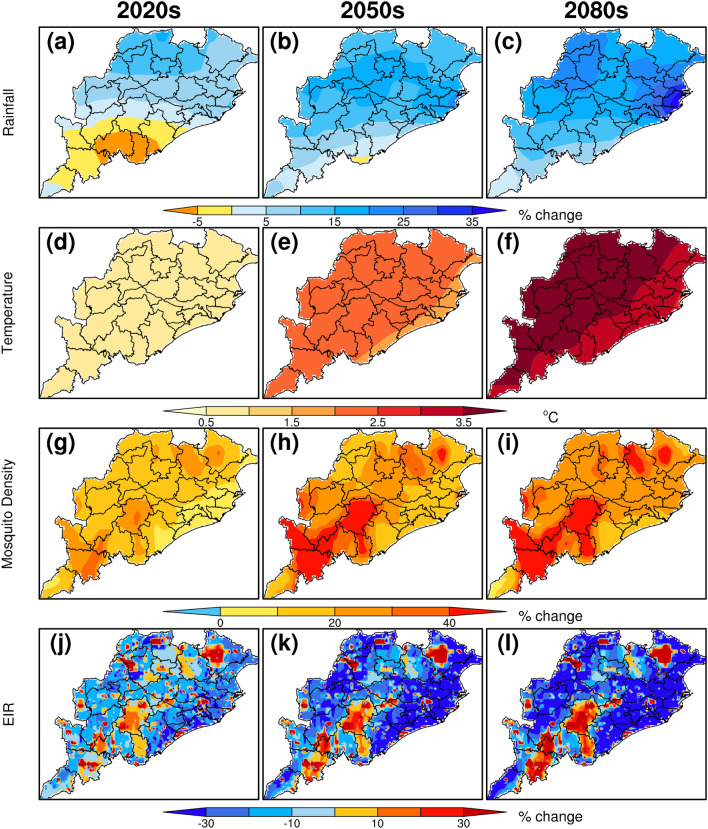
Projections of all the VECTRI simulated four parameters for the periods 2020s, 2050s and 2080s during summer monsoon season (June–September).

The percentage change in rainfall, temperature, mosquito density, and EIR during summer monsoon season (JJAS) for the projection periods 2020s, 2050s, and 2080s compared to the baseline period shows (Fig. 5a–c) that the rainfall over most of the Odisha is likely to increase by 10 to 40% for the periods 2020s, 2050s and 2080s. Odisha receives an average daily rainfall of 7–10 mm/day during the summer monsoon season. Results from future projections show that, there is an increase in rainfall by 5–15% over most parts of Odisha except over southern districts of Odisha where it may likely to decrease by 10–20% for the projection period 2020s with respect to the baseline period. A large percentage increase of 30–40% is projected by end of the century over north western and north eastern states of Odisha including the districts Cuttack, Jajpur, Kendrapara, Jagatsinghpur, Bhadrak, Balasore, Sambalpur, Bargarh, Jharsuguda, Sundargarh etc.

The mean temperature over Odisha during summer monsoon season shows about 25–30 °C with the maximum temperatures of about 29–30 °C over the eastern and northwest regions, and with the low temperature ranges between 25 °C and 26 °C over southern districts of Odisha. The projections of maximum temperature over most of the Odisha show an increase of 0.5–1.5 °C, 1.5–3 °C and 3–4 °C for the periods 2020s, 2050s and 2080s, respectively, with respect to baseline period (Fig. 5d–f).

The historical mean values of the mosquito density ranges between 0.01 and 0.05 per square meter in most of the Odisha region, with a maximum over the northeastern districts such as Cuttack, Puri, Kendrapara, Bhadrak, Balasore, Mayurbhanj Jajpur etc. Compared to baseline period, the future mosquito density (by end of the century) shows increase over most parts of Odisha by an average of 10–40% and it shows above 40% over few high elevated hilly regions with forest covers that includes the districts such as Koraput, Raygada,Nabarangpur,Kandhamal etc.(Fig. 5g–i).

The EIR values during the baseline period showing the range 0.5–4.8 ib /person/day, with the highest values above 3 ib/persons/day is seen over central, north and southern Odisha including few districts such as Kandhamal, Malkangiri, Kendujhar etc. The percentage change in EIR in 2020s, 2050s and 2080s shows an overall decrease of 5%, 13%, and 15%, respectively compared to the baseline period over Odisha as a whole but over few districts such as Mayurbhanj, Rayagada, Kandhamal and Koraput, EIR values are projected to increase by 10–30% by end of the century (Fig. 5j–l). These districts are mainly high-elevated and forested regions with remote rural tribal settlements. The risk of malaria prevalence continues to be high in those forested and remote rural districts of Odisha where the launching of several malaria control strategies in recent years have so far not improved the situation significantly^40^. In these forested areas of the hilly and mountainous type with tribal settlements where there is poor access to healthcare services and these communities are at high-risk to the disease outbreaks.

#### Projections during winter monsoon season

**Figure 6:**
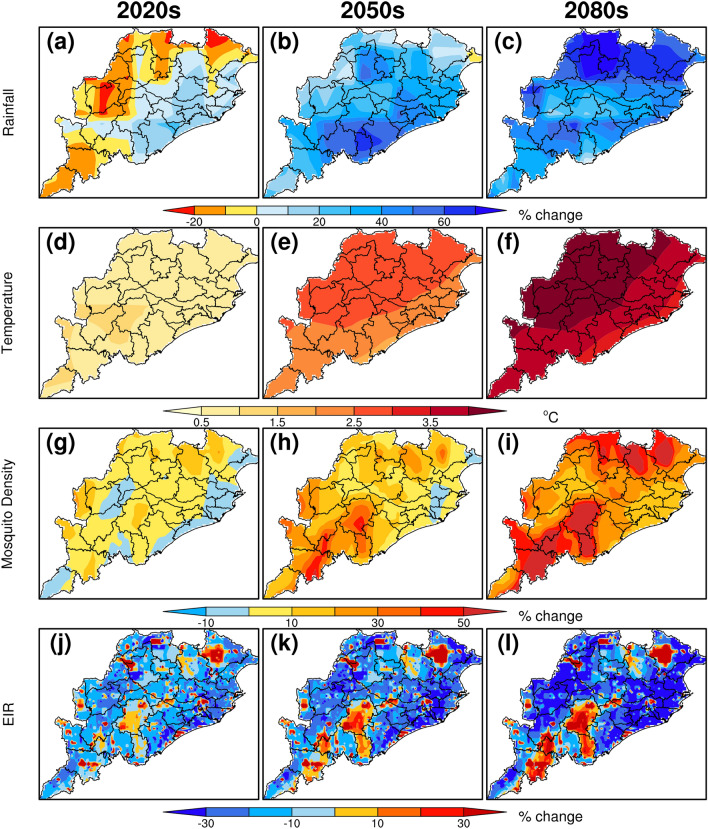
Same as Fig. 5 but for winter monsoon season (October-December).

The percentage change in rainfall, temperature, mosquito density, and EIR during winter monsoon season (OND) for the projection periods 2020s, 2050s and 2080s using CCSM4 global climate model under RCP8.5 scenarios are compared to the baseline period outputs. It is observed (Fig. 6a–c) that the future winter monsoon rainfall is increasing of about 50–75% (of about 25–50%) over coastal and northern districts of Odisha (over west, central and southern districts of Odisha) with respect to the baseline period. Compared to the west and south, the northern and coastal districts of Odisha are projected to have a higher rainfall by 2080s.

The mean winter temperature over the Odisha shows of about 21–25 °C during the baseline period, with the higher temperature over eastern regions of Odisha includes Puri, Cuttack, Khordha, Jajpur, Jagatsinghpur, Kendrapara districts. The projections of maximum temperature show an increase of 0–1 °C, 1–2 °C and 3–4 °C for the periods 2020s, 2050s and 2080s, respectively, over most of the Odisha with respect to baseline period (Fig. 6d–f). The percentage change in vector density is also projected to increase by about 10–60% in most of the region whereas the geographic distribution of the malaria vector shows shrinking in few of the coastal districts (Fig. 6g–i). Further, EIR values are projected to decrease by 13% by end of the century over Odisha as a whole. In most of the decrease in EIR remains within the range 10–30% but in few districts such as Kandhamal, Raygada, Mayurbhanj and Koraput, the EIR values are projected to increase by 30% by end of the century, which indicates a future increase in malaria transmission over these regions (Fig. 6j–l).

#### Projections of malaria transmission during warm and cold nights

**Figure 7:**
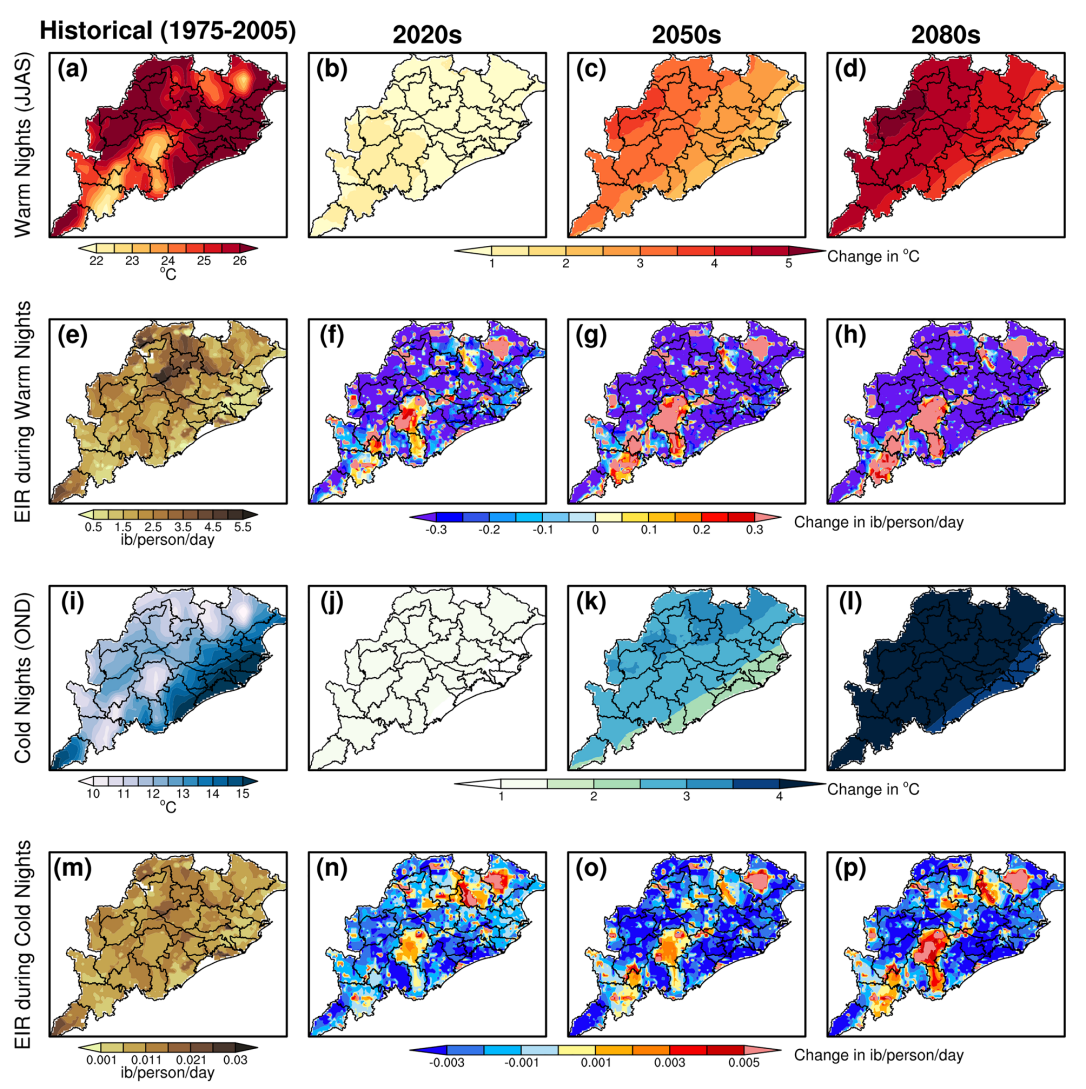
Projections of EIR during 2020s, 2050s and 2080s corresponding to the future changes in warm nights and cold nights.

Figure 7 shows the temperature and EIR values during warm nights (cold nights) of JJAS(OND) season for the historical period. Also, it shows the changes in future temperature and EIR during warm and clod nights of 2020s, 2050s and 2080s with respect to the historical period. Results suggest that warm night’s temperature (90th percentile of night temperature) remains between 24 and 26 °C over eastern and western Odisha and 22–24 °C over the central, southern and northern Odisha during the summer monsoon season of historical period. It is observed that future warming nights are likely to increase by 4–5 °C in most parts of Odisha by end of the century with respect to the baseline period. Further, temperature remains 10–12 °C over most of the Odisha during the cold nights (10th percentile of night temperature) of winter season except for the coastal belt where temperature remains 12–15 °C or above.

The warm nights (cold nights) temperatures show an increase of 0.9 °C (1.2 °C), 2.9 °C (2.8 °C) and 4.4 °C (4.3 °C) for the period 2020s, 2050s and 2080s with respect to the historical period, respectively. The projections of EIR during warm nights (cold nights) show a decrease of 5.51% (9.05%), 7.71% (15.58%) and 12.07% (8.92%) for the period 2020s, 2050s and 2080s with respect to the historical period respectively. It can be noticed that malaria transmission is likely to reduce in future over most of the Odisha regions with the increase in future warm and cold nights temperatures. However, some pockets in southern, central and northern parts of Odisha that includes the districts like Kandhamal, Rayagada, Koraput, Gajapati, Kendujhar and Mayurbhanj districts show an increase in malaria transmission with the future increase in warm and cold nights temperatures.

## Discussions

On applying the Mann–Kendall trend test on future trends of malaria transmission in terms of EIR data during 2020s, 2050s and 2080s, it is seen that decreased trend of EIR during OND season is significant at 0.1 level as the p values are less than the significance level α (alpha) = 0.1 and the null hypothesis is rejected (R-H0). Further, Decreasing trend in future projections of EIR during JJAS season over Odisha region is not significant at 90% confidence level as the p value are greater than the significance level α (alpha) = 0.1 and the null hypothesis is accepted (A-H0). The details of the p values and test interpretation is given in Table 3.

**Table 3:** Results from the Mann–Kendall trend test using EIR data for the whole Odisha during JJAS and OND season.

Season	Trend Result	2020s	2050s	2080s
JJAS	Slope	0.011	− 0.004	0.006
	*P*-value	0.124	0.668	0.372
	Test Interpretation	A-H0	A-H0	A-H0
OND	Slope	0.0001	0.014	0.006
	*P*-value	0.971	0.003	0.063
	Test Interpretation	A-H0	R-H0	R-H0

Our results about malaria reduction in most of the regions in Odisha are consistent with few previous studies. For example, one of the studies by Dhiman et al. (^29^) suggests that there may be reduction in malaria transmission windows over Odisha due to high temperatures in future). In another study by^51^ using VECTRI model simulations driven by many global climate model data sets, malaria transmission is likely to reduce over many eastern regions of India including Odisha. Past studies on malaria transmission carried out so far over Odisha region are mainly focused on observation period. For example, one such study carried out by Pradhan et al.^41^ reveals that there is a significant decrease in malaria incidence over Odisha during the period 2003–2013. In one of our previous papers, we have used VECTRI dynamical model for investigating malaria transmission dynamics over Odisha during the past period 1975–2005 and the results suggest that the intensity of malaria transmission is found to be higher in some of the north, central and southern districts of Odisha, with forest or mountainous ecotypes where the mosquito populations and the number of infective bites are more^43^. This is the first ever study over the Odisha region that focuses future malaria transmission on a spatial and temporal scale using a dynamical malaria model.

## Conclusions

In this study, we have used VECTRI malaria model to assess the effect of climate change on malaria transmission over the Odisha state. The results show that change in temperature and rainfall in future may influence the potential changes in the malaria transmission in Odisha. The CCSM4 model appears to project future increases in temperature and rainfall over most parts of Odisha and it is found that the future changes in increasing temperature and rainfall could potentially increase mosquito density and decrease EIR by the end of the century under RCP8.5 scenarios. The average maximum surface temperature is projected to increase by 3–4 °C by end of the century over Odisha as a whole. Our findings suggest that spatial and temporal distribution of malaria transmission in Odisha during both the monsoon seasons (summer and winter) may decrease, with an increase in temperature of 3–4 degree and increase in rainfall of 20 to 40% by end of the century with respect to the present day climate. Similar results are seen for the future changes in warm nights(cold nights) temperatures by 4.4 °C(4.3 °C) where malaria transmission is likely reduce by 12.07% (8.92%) by end of the century with respect to the baseline period, respectively. In particular, the decreasing trend in malaria transmission during the post monsoon season (OND) is statistically significant (Mann–Kendall trend test).

Knowing that fact that malaria transmission dynamics depends on various factors like climate change, socioeconomic conditions, public health care systems, this modelling approach of using projection of change in malaria transmission based on temperature and rainfall may strengthen and motivate various research communities as well as government administrators for the climate driven disease modeling over Odisha. This approach of using climate change projections and its impact on malaria dynamics may be useful to take actions against malaria eradications by the decision makers and the local administrators in the state. Although this study presents the results from one GCM (CCSM4) projection, our future study will focus on evaluating an accuracy of many such GCMs with all possible RCPs and with different uncertainty assessments over the region.

## Data Availability

Land surface topographic information were extracted from the SRTM (Shuttle Radar Topographic Mission) dataset (http://srtm.csi.cgiar.org/srtmdata/) available at ~ 30-m resolution to represent the study area (Fig. 1) with respect to the height from the sea level and to know about the plain stations areas in the study region. The CCSM4 global climate model data sets are available ESGF data portal (https://esgf-node.llnl.gov/search/cmip5/) which is freely accessible. We have used different atmospheric variables such as daily maximum temperature, daily minimum temperature and daily rainfall data from India Meteorological Department. The description of the data used in present study are given in methodology section.
